# Features associated with cognitive impairment and dementia in a community-based sample of illiterate elderly aged 75+ years: the Pietà study

**DOI:** 10.1590/S1980-57642014DN82000007

**Published:** 2014

**Authors:** Henrique Cerqueira Guimarães, Jorge Luiz Cascardo, Rogério Gomes Beato, Maira Tonidandel Barbosa, Thais Helena Machado, Mariana Alves de Almeida, Simone Rios Fonseca Ritter, Karina Braga Gomes Borges, Antonio Lucio Teixeira, Paulo Caramelli

**Affiliations:** Behavioral and Cognitive Neurology Research Group , Faculty of Medicine, Federal University of Minas Gerais, MG, Brazil.

**Keywords:** aging, cognitive impairment, dementia, elderly, illiteracy

## Abstract

A higher level of educational attainment constitutes a protective factor against cognitive decline in the elderly. Nevertheless, the elements underpinning this association are yet not fully understood.

**Objective:**

The primary aim of this study was to compare cognitively impaired illiterate elderly subjects with cognitively preserved counterparts, according to demographics, comorbidities, lifetime habits and APOE genotype.

**Methods:**

This is a cross-sectional analysis of the illiterate subset of participants (n=174) from the Pietà study, a community-based survey of successful brain aging conducted in Caeté (MG), Brazil. Subjects were categorized into three diagnostic groups: cognitively normal (CN), cognitive impairment no-dementia (CIND) and dementia. The groups were then compared according to selected variables.

**Results:**

Subjects with dementia were older and had an increased prevalence of reported stroke or transient ischemic attack. The three groups did not differ in relation to demographics, prevalence of comorbidities, socioeconomic level, previous occupation profile and APOE-ε4 allele frequency. Qualitatively evaluated lifetime habits, such as alcohol consumption, smoking and physical activity engagement were also similar across groups.

**Conclusion:**

No associations were found between cognitive impairment/dementia and the variables evaluated in this community-based sample of illiterate elderly.

## Abstract (pt)

Um alcance educacional mais elevado constitui um fator protetivo contra o declínio cognitivo em idosos. Todavia, os elementos subjacentes a esta associação ainda são pouco compreendidos.

**Objetivo:**

O principal objetivo deste estudo foi comparar indivíduos analfabetos com comprometimento cognitivo com analfabetos cognitivamente normais, de acordo com aspectos demográficos, morbidades, hábitos de vida e genótipo APOE.

**Métodos:**

Este é um estudo transversal da amostra de participantes analfabetos (n=174) do Estudo Pietà, um levantamento de base comunitária sobre envelhecimento cerebral bem sucedido, conduzido em Caeté (MG), Brasil. Os sujeitos foram categorizados em três grupos diagnósticos: cognitivamente normais, comprometimento cognitivo não demência e demência. Os grupos foram então comparados conforme variáveis selecionadas.

**Resultados:**

Indivíduos com demência eram mais idosos e apresentaram uma maior prevalência de relato de acidente vascular encefálico ou ataque isquêmico transitório. Os três grupos não se mostraram diferentes em relação à demografia, prevalência de comorbidades, nível socioeconômico, perfil ocupacional prévio e frequência do alelo APOE-ε4. A avaliação qualitativa de hábitos de vida, como o consumo de bebida alcoólica, fumo e engajamento em atividade física também foi semelhante entre os grupos.

**Conclusão:**

Não encontramos associações entre comprometimento cognitivo/demência e as variáveis investigadas nesta amostra comunitária de idosos analfabetos.

## INTRODUCTION

Large epidemiological surveys of cognitive impairment and dementia in the elderly have consistently found that a higher level of educational attainment is associated with a lower prevalence of dementia, and also suggest a possible early dementia onset in low educated subjects.^1^ The neurobiological underpinnings thought to mediate the relationship between education and protection against cognitive impairment are not yet fully understood. An influential theory, the cognitive reserve hypothesis, states that education promotes an increased number and stronger neuronal connections, which collectively ensure resilience against neuropathological load.^2^ However, it is reasonable to hypothesize that a higher educational level also provides expanded access to information, and potentiates professional achievement, which in turn are both associated with a healthier life style and facilitated access to better health care services, conferring further protection in more educated subjects against vascular disease.^3^

Most data regarding the cognitive reserve hypothesis is derived from cohorts of subjects followed in developed countries, which typically include participants with higher educational level.^4,5^ There is much less data regarding dementia prevalence and its related factors in very low educated populations, especially in illiterate subjects. In a recent report from the Brazilian Brain Bank Study, Farfel and colleagues showed that even a few years of education, when compared to illiteracy, provided resilience against neuropathological burden, in accordance with the cognitive reserve hypothesis.^6^ The group's results are especially salient since they were independent of cerebrovascular disease pathology. Albeit sound, these data are retrospective in nature, and were drawn from a selected population of the largest city in Brazil, which might not constitute a broad range representation of lower income settings in the country.

In order to contribute to this important issue, herein we report from a community-based sample of Brazilian elderly with very low education, a cross-sectional analysis from the Pietà study, comparing demographics, life style habits, comorbidity features, and APOE genotype between cognitively impaired and cognitively preserved illiterate subjects.

## METHODS

The target population of this report comprised the illiterate subset of participants (n=175) from The Pietà Study, a community-based survey of successful aging carried out in Caeté (MG), Southeast Brazil, between October, 2008 and March, 2009 (first wave). Detailed methodology has been described previously.^7^ Briefly, the study invited all the city's inhabitants aged 75 years or older to participate, and those who agreed gave written informed consent. The study was approved by the university ethics committee. The evaluation procedures were accomplished through three stages: at phase 1, participants (n=639) were submitted to an in-home structured interview; at phase 2 they underwent a comprehensive clinical evaluation within an outpatient setting and, if indicated, subjects were then submitted to a neuropsychological assessment; at phase 3 a large subset (n=358) of the participants provided morning fasting blood samples for research purposes, including APOE genotyping, while a smaller subset of participants (n=188) underwent a standardized head magnetic resonance imaging protocol.

In phase I, participants answered a detailed and structured questionnaire including: socioeconomic level (from A=higher to E=lower), quality of life evaluation (WHOQOL-OLD), global functional information, mobility, current and previous physical activity (Baecke Habitual Physical Activity Questionnaire), leisure activities, information on religious beliefs and attendance of religious cults, smoking and drinking habits, sleep habits, nutritional information, and self-reported information on hearing and visual function. Whenever possible, information was quantitatively assessed. Previous occupation was classified into one of the following categories:^8^

[1] rural unskilled manual workers;
[2] urban unskilled manual workers (menial and repetitive tasks);
[3] skilled manual workers (specific tasks that require training);
[4] routine non-manual employees and self-employed workers; and
[5] intellectuals, administrators and higher-level technicians.

In phase II, subjects were submitted to a thorough functional, clinical, psychiatric and neurological evaluation, including the Functional Activities Questionnaire (FAQ), the motor section of the Unified Parkinson's Disease Rating Scale (UPDRSm), the Mini International Neuropsychiatric Interview, Geriatric Depression Scale (GDS), and a Brief Cognitive Screening Battery (BCSB), consisting of the Mini-Mental State Examination (MMSE), animal category semantic fluency test, and picture drawings memory test (PDMT). Those regarded as having possible cognitive impairment plus a subset of putative cognitively healthy controls were further submitted to a comprehensive neuropsychological evaluation with the following instruments: Rey Auditory Verbal Learning Test, naming and praxis tests from the CERAD (Consortium to Establish a Registry for Alzheimer's Disease) protocol, phonemic verbal fluency tasks (FAS), Frontal Assessment Battery and the Mattis Dementia Rating Scale.

Participants' performances were judged pursuant to four levels of educational attainment, namely, illiterates (n=175), 1 to 3 years (n=247), 4 to 7 years (n=177) and above 7 years of formal education (n=44), in agreement with previous Brazilian reports using these same brief tests in low educated subjects.^9,10^ A reference cut-off on the MMSE was set at the 25^th^ percentile from the epidemiological survey of dementia in a Brazilian community,^11^ previously carried out in Catanduva, in accordance with the baseline performance of the participants that remained free of dementia in a three-year follow-up.^12^ A cut-off of 5 points on the FAQ was set do define a significant level of functional impairment.^13^

Even though reference values in specific tests and evaluations were used to judge participants' performance, diagnostic formulations were not constrained by cut-off parameters. The presence of cognitive and functional impairment was based on careful group discussions and consensus agreement among the examining physicians involved in the collecting of clinical data, taking into account all the evaluations available, education, prior occupation, visual or hearing deficits, and other relevant information. Dementia diagnosis was based on Diagnostic and Statistical Manual of Mental Disorders, 4^th^ edition [DSM-IV] criteria.^14^ Subjects considered to have cognitive impairment according to consensus agreement but that performed below the threshold on the FAQ were grouped as "cognitive impairment no-dementia (CIND)". Subjects without any evidence of cognitive and functional impairment were considered cognitively normal (CN).

**Statistical analysis.** One of the 175 illiterate participants was excluded from the analysis because of extensive missing data. Two types of comparison were performed: a first comparison among the three distinguished groups (CN, CIND and dementia); and the second between cognitively impaired groups (CIND + dementia) and CN subjects. According to the D'Agostino-Pearson test, continuous variables showed non-normal distribution. Hence, comparisons between groups and among them were analyzed with the Mann-Whitney and Kruskal-Wallis tests, respectively. Categorical data were analyzed according to the Chi-square test. Statistical significance was set for p values ≤0.05.

## RESULTS

Among the 174 illiterate participants, there was a predominance of women (70.1%), and a mean age of 80.9±4.9 years. These demographic findings are very similar to those for the whole cohort, previously reported.^7^ In accordance with cognitive diagnoses, this sample was categorized into three groups as follows: 46% CN, 22.4% diagnosed as CIND and 31.6% as having dementia.

According to Table 1, the mean age of the participants was slightly but significantly higher in the dementia group (82.8±5.7 years; p=0.02). The groups were clearly distinguished regarding cognitive performance on the brief screening tests (p<0.0001for all comparisons). As expected, the dementia group had worse performance on the functional evaluation (FAQ) in comparison with CN and CIND groups (p<0.0001).

**Table 1:** Age and performance on brief cognitive tests and functional evaluation.

	CN			CIND			Dementia			CI			Analysis 1	Analysis 2
	n=80			n=39			n=55			n=94				
	Mean	SD		Mean	SD		Mean	SD		Mean	SD		p-value	p-value
Age (y)^c^	79.9	4.6		80.5	3.6		82.8	5.7		81.8	5		0.02	0.047
Animal category fluency^a,b,c^	10.9	2.9		9.6	3		6.4	3		7.9	3.4		<0.0001	<0.0001
PDMT - delayed recall^a,b,c^	7.4	1.4		5.2	1.8		3.7	2.7		4.4	2.5		<0.0001	<0.0001
MMSE^a,b,c^	19.8	2		15.8	2.4		12.7	3.9		14.1	3.7		<0.0001	<0.0001
FAQ^b,c^	2.4	4.1		2.5	5		16.8	9.8		10.9	10.8		<0.0001	<0.0001

CN: cognitively normal; CIND: cognitive impairment no-dementia; CI: dementia+CIND combined group; Analysis 1: comparison among groups; Analysis 2: comparison between CN and CI groups; y: year; PDMT: Picture drawings memory test; MMSE: Mini-mental state examination; FAQ: Functional activities questionnaire;

a CN ≠ CIND;

b CIND ≠ Dementia;

c Dementia ≠ CN.

Table 2 shows the categorical variables analyzed according to cognitive syndrome diagnosis. There were no significant differences among groups regarding sex or socioeconomic level distribution. On the "previous occupation" analysis, no participants were categorized as urban non-skilled manual worker in the CIND group, and this difference was statistically significant (p=0.02) in comparison with the CN and dementia groups. No associations were found between reported comorbidities and the cognitive outcomes evaluated, although an increased prevalence of reported stroke or transient ischemic attack (TIA) was identified in the dementia group (31.7%; p=0.006). Negative results were also found for association between cognitive group category and current depressive syndrome. Life time habits, such as drinking, smoking and physical activity were also found not to be associated with diagnostic syndrome. As could be reasonably expected, no participants with dementia diagnosis reported current physical activity engagement (p=0.006).

**Table 2:** Sociodemographics, comorbidities, life time habits and APOE genotype.

			CNn=80	CINDn=39	Dementian=55	CIn=94	Analysis 1p-value	Analysis 2p-value
Gender (M/F)			27/53	11/28	14/41	25/69	ns	ns
Life time occupation category (%)	1- rural unskilled manual worker		41.8	59.0	38.8	47.7	ns	ns
	2- urban unskilled manual worker^a,b^		19.0	0	13.0	7.6	0.02	0.047
Socioeconomic level	C		15.9	18.9	15.4	17.1	ns	ns
	D		66.7	48.6	69.2	59.2	ns	ns
	E		17.4	32.4	15.4	23.7	ns	ns
Comorbidity (%)	Diabetes mellitus		19.2	13.2	20.0	17.0	ns	ns
	Hypertension		77.5	74.4	78.4	76.7	ns	ns
	Reported stroke or TIA^b,c^		10.0	5.1	31.7	16.7	0.006	ns
	Reported MI		3.8	5.1	6.1	5.7	ns	ns
	Reported cancer		8.9	5.1	2.0	3.4	ns	ns
	Reported depression		15.0	13.2	16.0	14.8	ns	ns
	Current depressive disorder		15.0	15.4	15.7	15.6	ns	ns
Life time habits (%)	Smoking habit	current	9.3	7.9	16.0	12.5	ns	ns
		any time	39.7	36.8	35.6	36.1	ns	ns
	Drinking habit*	current	10.9	7.9	7.3	7.6	ns	0.075
		any time	57.1	28.9	33.3	33.7	0.10	0.07
	Physical activity habit	current^b,c^	19.2	18.4	0	8.2	0.006	0.079
		any time	22.9	12.8	21.6	16.7	ns	ns
	APO-e4 allele carrier (n=78)		24.4	23.8	43.8	32.4	ns	ns

CN: cognitively normal; CIND: cognitive impairment no-dementia; CI: dementia+CIND combined group; Analysis 1: comparison among groups; Analysis 2; comparison between CN and CI groups. TIA: Transient ischemic attack; MI: Myocardial infarction;

a CN ≠ CIND;

b CIND ≠ Dementia;

c Dementia ≠ CN.

* Only one participant reported features consistent with alcohol abuse.

## DISCUSSION

In this cross-sectional analysis of the illiterate subset of participants from the Pietá Study, hardly any features were found to be associated with cognitive impairment sensu lato, or among the distinguished diagnosed groups, i.e., CN, CIND or dementia. The only consistent significant result was the expected association between dementia/cognitive impairment and an older age, and the association between dementia and a clinical history of stroke or TIA.

In contrast to the findings of Scazufca and colleagues,^15^ we disclosed no association between socioeconomic class, previous occupation and dementia. Compared to the cited study, we reported on a smaller sample but studied a specific educational stratum, which might better account for the hard-to-control interactions between low education, low socioeconomic status and unskilled occupations. Indeed, our result is in accordance with two other community-based studies conducted in Brazil. The epidemiologic survey of dementia in Catanduva found no association between the diagnosis and socioeconomic class.^11^ Moreover, within the subset of participants without formal education from the São Paulo and Ribeirão Preto studies of cognitive and functional impairment, an outcome defined as MMSE performance below the 15^th^ percentile was also not associated with socioeconomic class.^16^ We should acknowledge, however, that the analysis herein reported might be constrained by a floor effect, since the wealthiest classes (A and B) were not represented in this illiterate sample.

No reported comorbidity was associated with the cognitive groups, except a higher frequency of reported cerebrovascular events in the dementia group. However, since these data are derived from a cross-sectional analysis, it is not possible to conclude whether these possible cerebrovascular syndromes, reported as stroke or TIA, are causally associated with dementia or merely a symptom of their underlying brain disease. Modeling relationships between multimorbidity and cognition within a statistical framework is a pain-staking endeavor. Birth cohorts of current elderly have strongly suggested that cognitive function in childhood can play an important role in defining some lifetime habits, such as smoking and caffeine consumption in adulthood.^17,18^ Furthermore, these studies also show that cognitive performance during childhood is a major determinant of cognitive performance in oldest ages.^19^ Hence, cross-sectional analysis probing associations between comorbidities and cognition are probably not the best strategy to tap into this issue. This will require prospectively designed long-term studies, aiming at studying comorbidity in adulthood and its' consequences years after in older age.^20^ The striking negative results regarding depressive disorder prevalence and cognitive performance stand at odds with previously reported findings,^16,21^ and highlight the importance of continuing research into illiteracy and elderly neuropsychiatric disorders. The lack of findings regarding lifetime habits and cognitive syndrome are constrained by the qualitative nature of the analysis, extracted from the extensive Phase 1 interview. An interesting finding, with a trend towards statistical significance, suggested an increasing prevalence of lifetime drinking habits in the cognitively preserved group. Indeed, a few recent reports document an association between low and moderate alcohol intake in middle age and cognitive preservation, especially in women.^22-24^ There was clearly a large number of APOE-e^4^ allele carriers in the dementia group, although this result did not reach the statistical threshold. We speculate that this finding would have reached significance in a larger study sample.

Some limitations of our study must be recognized. Although a large number of illiterate subjects were evaluated within a comprehensive protocol, the sample remained insufficient to draw robust results. The cross-sectional design cannot rule out the possibility of a survival bias, which could partially explain the absence of an association between cognitive outcomes and established risk factors for cerebrovascular disease. Also, quantitative results regarding drinking, smoking and physical activity habits were not given, which may have potentially modified the present discussion. Another important limitation pertains to the questionable applicability of the extensive structured interview from Phase 1 to illiterate subjects.
